# HMFT: Hyperspectral and Multispectral Image Fusion Super-Resolution Method Based on Efficient Transformer and Spatial-Spectral Attention Mechanism

**DOI:** 10.1155/2023/4725986

**Published:** 2023-03-01

**Authors:** Baojun Qiao, Binghui Xu, Yi Xie, Yinghao Lin, Yang Liu, Xianyu Zuo

**Affiliations:** ^1^Henan Key Laboratory of Big Data Analysis and Processing, School of Computer and Information Engineering, Henan University, Kaifeng 475000, China; ^2^Henan Engineering Laboratory of Spatial Information Processing, Henan University, Kaifeng 475000, China

## Abstract

Due to the imaging mechanism of hyperspectral images, the spatial resolution of the resulting images is low. An effective method to solve this problem is to fuse the low-resolution hyperspectral image (LR-HSI) with the high-resolution multispectral image (HR-MSI) to generate the high-resolution hyperspectral image (HR-HSI). Currently, the state-of-the-art fusion approach is based on convolutional neural networks (CNN), and few have attempted to use Transformer, which shows impressive performance on advanced vision tasks. In this paper, a simple and efficient hybrid architecture network based on Transformer is proposed to solve the hyperspectral image fusion super-resolution problem. We use the clever combination of convolution and Transformer as the backbone network to fully extract spatial-spectral information by taking advantage of the local and global concerns of both. In order to pay more attention to the information features such as high-frequency information conducive to HR-HSI reconstruction and explore the correlation between spectra, the convolutional attention mechanism is used to further refine the extracted features in spatial and spectral dimensions, respectively. In addition, considering that the resolution of HSI is usually large, we use the feature split module (FSM) to replace the self-attention computation method of the native Transformer to reduce the computational complexity and storage scale of the model and greatly improve the efficiency of model training. Many experiments show that the proposed network architecture achieves the best qualitative and quantitative performance compared with the latest HSI super-resolution methods.

## 1. Introduction

Hyperspectral imaging can capture images of the same scene at different wavelengths simultaneously. Its rich spectral features are of great importance in the field of remote sensing [1]. HSI has been applied to tracking [2], classification [3–5], segmentation [6], and clustering [7], and the results are significantly improved. Compared with conventional images (e.g., colour or grayscale images), HSI contains richer spectral information of real scenes. However, images with high spatial resolution and high spectral resolution cannot be obtained simultaneously due to the limitations of existing imaging sensors. However, it is easier to obtain RGB (red, green, blue) or panchromatic images with higher spatial resolution but lower spectral resolution [8] for conventional cameras. Therefore, fusing low-resolution hyperspectral images (LR-HSI) and multispectral high-resolution images (HR-MSI) is an effective way to solve the hyperspectral super-resolution problem. This process is often referred to as HSI super-resolution or HSI fusion.

Currently, the fusion problem is formulated as an image restoration problem [8–18]. The method follows a physical degradation model where the input LR-HSI and HR-MSI images are considered as spatially degraded observations and spectrally degraded observations of potential HR-HSI, respectively. The observation model is expressed as

(1) $$\begin{array}{c} Y=BS\text{,}\\ Z=RX\text{,} \end{array}$$

where *Y* ∈ *ℝ*^*S*×*hw*^, *Z* ∈ *R*^*s*×*HW*^, and *X* ∈ *R*^*S*×*HW*^ represent the two-dimensional matricesof LR-HSI, HR-MSI, and HR-HSI after expansion along the third dimension, respectively. *h* and *w* refer to the width and height at low resolution, and *H* and *W* refer to the height and width at high resolution, and *s* and *S* represent the number of spectral bands at multispectral and hyperspectral levels, respectively. In addition, ∈ *ℝ*^*HW*×*HW*^, *S* ∈ *ℝ*^*HW*×*hw*^, and *R* ∈ *ℝ*^*s*×*S*^ are the blur matrix, subsampling matrix, and spectral response matrix, respectively. Based on the observation model (1), many methods have been proposed and achieved good performance.

HSI super-resolution has a large-scale factor in both spatial and spectral domains, and it is a highly ill-defined problem. Therefore, it is vital to integrate a priori information to constrain the solution space. Reference [19–21] uses the prior knowledge that the spatial information of HR-HSI can be sparsely represented under the dictionary trained by HR-HSI [22]. A local spatial smooth prior for HR-HSI images is assumed, which is encoded into the optimization model using total variational regularization. The low-rank characteristics of the spectrum are utilized to reduce spectral distortion [23]. Although valid for some applications, its rationality depends on subjective prior assumptions about the unknown HR-HSI. However, the HR-HSI images collected in real scenes are highly spatially and spectrally diverse. This traditional learning method cannot adapt to different HSI image structures.

In recent years, deep learning has performed better than traditional methods in many computer vision tasks [24]. Satisfactory performance is also achieved in HSI/MSI image fusion field. An enhanced deep learning model is proposed by combining the observation model with low-rank prior information of HSI spectral. For example, Dian et al. [25] have designed a deep model to learn the prior information inside the image through deep CNN. This learned prior information can be combined with the traditional regularization model to obtain better image features than the single regularization model. In the study of [26], a multiscale fusion model is proposed, which adaptively adapts features in combination with the attention model, showing the ability to retain spectral information andspatial details, thus obtaining the most advanced HSI super-resolution results. Compared with traditional methods, the CNN-based approach is significantly improved, but it still suffers from its own drawbacks. For example, CNN-based methods focus on fine architecture design, and the model is usually complex. Secondly, CNN pays more attention to local features, and the model effect of long-term dependence and global features is poor.

Recently, we noticed that Transformer [27] and its various variants have achieved remarkable achievements in natural language processing and advanced computer vision tasks. Transformers have also been introduced into low-level vision tasks due to their excellent performance. For example, Chen et al. [28] proposed a multitask large-scale model IPT based on the original Transformer for image super-score. Liang et al. [29] proposed the SwinIR model based on the Swin-Transformer to solve the problem of image restoration. Both methods are aimed at the naturalimage restoration problem and lack the properties of HSI. Subsequently, Hu et al. [30] proposed a pixel-level Transformer model FuseFormer to solve the HSI/MSI fusion problem. However, the network's ability to extract local features is insufficient, and the restoration of spatial details is mainly concerned without considering spectral features.

Based on the previous factors, we propose a simple and efficient hybrid network model HMFT based on a Transformer to solve the problem of hyperspectral image fusion. Specifically, HMFT is mainly composed of spectral information extraction and spatial information extraction. (1) In spectral information, LR-HSI is up-sampled to a high-resolution scale and directly transmitted to the end of the network through a long connection, so as to retain the spectral information contained therein to the maximum extent; (2) in spatial information, through clever design, the spatial details and remaining spectral information are extracted by fully combining the advantages of CNN, which pays more attention to local features, and Transformer, which pays more attention to global features. In addition, the feature segmentation module (FSM [31]) is added to reduce the time and space complexity of the network, and the Convolution Block Attention Module (CBAM [32]) is added to explore the correlation between the spectral to promote spatial enhancement and spectral consistency. Finally, the extracted spectral information and spatial information are fused to generate HR-HSI. In summary, the main contributions of this article can be summarized as follows:

- A novel model HMFT based on Transformer is proposed to solve the super-resolution problem of hyperspectral images. The self-attention mechanism of the Transformer can capture the global interaction between contexts and make up for the disadvantage that CNN only focuses on local features. We combine the advantages of both to extract rich feature information.
- Considering the huge amount of hyperspectral data, the native Transformer needs a lot of memory and calculation, and the model is difficult to train. Therefore, the feature split module (FSM) is introduced to replace the native Transformer self-attention calculation method to reduce the space and time complexity of the model.
- Experimental results on three different datasets demonstrate that our proposed network model HMFT is effective and generalizes well compared to previous state-of-the-art methods.

## 2. Related Work

### 2.1. Deep CNN

Deep CNN-based learning methods [33–45] have achieved good performance in the field of image SR. Yang et al. [39] proposed the PanNet network model, which uses ResNet as the feature extraction backbone network. In particular, the network is trained with high-frequency information of images, which can reduce the network training pressure and retain more high-frequency information of images, while enhancing the generalization ability of the model. MHFnet [34] defines the fusion task as an optimization problem. It combines the degradation model of the hyperspectral image with the spectral low-rank prior of HSI to construct the algorithm model. Different from the traditional optimization algorithm solution method, they extend the near-end iterative algorithm to the CNN network model to learn the near-end operator and model parameters. Hu et al. [26] designed a multiscale fusion model HSRnet to extract spatial information of different scales and introduce an attention mechanism to make the network focus on important components of the image and suppress noise. Although all the previous methods achieve good results, the fact that CNN networks are limited by the size of the convolutional kernels cannot be ignored.

### 2.2. Vision Transformer

In recent years, the natural language processing model Transformer [27] has been gradually applied in the field of image super-resolution with its excellent performance. Chen et al. [28] proposed a multitask model IPT, which extracts image global information by stacking multiple native Transformer modules to solve low-level vision tasks. Liang et al. [29] proposed the SwinIR model, which uses residual Swin Transformer blocks (RSTB) as the basic unit to build a deep feature extraction network to solve the single image SR problem. Hu et al. [30] proposed the FuseFormer fusion model, which uses each pixel of the hyperspectral image as the input of the Transformer module to construct a pixel-level end-to-end mapping network. The Transformer module can significantly improve the performance of the network thanks to its advantage of building long-term dependencies on images. However, due to the huge parameter scale and high GPU performance consumption, it is rarely used in the field of hyperspectral image fusion.

## 3. Methodology

This section describes HMFT in detail. The purpose is to learn an end-to-end mapping function *F*_*θ*_(∙) with parameters *θ* by fully mining the spatial andspectral information between low-resolution hyperspectral image LR-HSI, high-resolution multispectral image HR-MSI, and ground truth HR-HSI. Finally, an image with high resolution and hyperspectral characteristics is reconstructed through

(2) $$\begin{array}{c} I=F_{\theta }\left(y,z\right)\text{,} \end{array}$$

where *y* ∈ *ℝ*^*H*×*W*×*s*^ and *z* ∈ *ℝ*^*h*×*w*×*S*^ are the HR-MSI and the LR-HSI, respectively. *I* represents the fusion result and *θ* represents the entire network parameter, which can also be regarded as implicit prior knowledge.

### 3.1. Network Architecture

**Figure 1:**
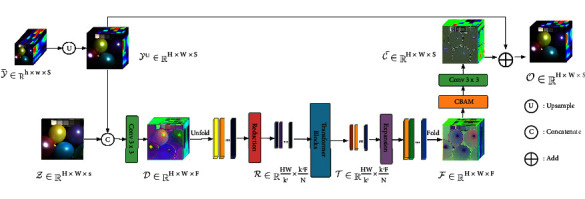
Schematic diagram of the overall network architecture (HMFT).

Figure 1 presents the overall schematic diagram of the HS/MS fusion network-based super-resolution model HMFT. The network takes LR-HSI and HR-MSI as input and finally outputs a HR-HSI. The network is mainly divided into upper and lower parts. The upper part is mainly composed of an upsampling module and a long residual connection, which is used to preserve the spectral information in LR-HSI to the greatest extent, and the lower part is composed of convolution layer, Efficient Transformer layer, and Spatial-Spectral Attention Module, which is used to preserve the spatial information and remaining spectral information.

### 3.2. Input of Transformer

As Figure 1 shows, the network takes LR-HSI *y* ∈ *ℝ*^*h*×*w*×*S*^ and HR- MSI *z* ∈ *ℝ*^*H*×*W*×*s*^ as input. The *y* is first upsampled to the same scale as *z* using a bicubic linear interpolation algorithm to obtain *y*^*u*^, followed by stitching with HR-MSI along the spectral dimension, immediately followed by a 3 × 3 convolutional layer, which works well for early visual processing and is more stable and optimal for extracting shallow spatial-spectral feature information [46].

(3) $$\begin{array}{c} D=Conv\left(\left[y^{u},z\right]\right)\text{.} \end{array}$$

The native Transformer [47] divides the original image into nonoverlapping blocks and then stretches them into a one-dimensional vector, while adding positional embeddings to represent the positional relationships between patches. Since our test data comes from images of various sizes in different scenes, there is a parameter mismatch. Considering the previous reasons, the positional embedding is removed and the unfolding technique is used to manipulate the feature map *D* ∈ *ℝ*^*H*×*W*×*F*^. The partitions of the “Unfold” operation are sequential and it automatically reflects the information about the location of each patch [31]. In detail, feature map *D* ∈ *ℝ*^*H*×*W*×*F*^ is unfolded (by kernel = stride = *k*) to a patch sequence, i.e., *d*_*i*_ ∈ *ℝ*^*k*^2^×*F*^, *i*={1, ⋯, *N*}, where *N*=(*HW*/*k*^2^) is the total number of the 1-D features. After that, those *ⅆ*_*i*_ are sent to the transformer module for further processing.

### 3.3. Efficient Transformer Blocks

**Figure 2:**
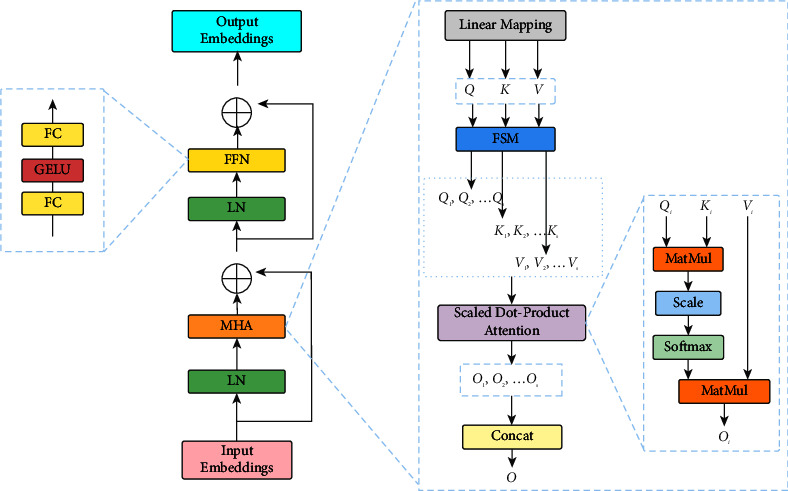
The transformer encoder module used in the network.

This part consists of several Transformer encoder modules. As illustrated in Figure 2, a single encoder block mainly consists of an efficient multihead self-attention module (EMHA [31]) and multilayer perception (MLP). Meanwhile, layer normalization (Norm [48]) and residual connections are interspersed.

Let's suppose that the input features *P* is *B* × *N* × *C*. Due to the large number of hyperspectral image bands, the input features dimension *C* after patch division is too high, which will lead to too many network training parameters, and the model is easy to fall into overfitting, making it difficult to train the network. Therefore, we add a reduction layer to reduce the dimension of input features by *n* times, where the reduction layer includes a full-join operation. Then for input features, *P* ∈ *ℝ*^*B*×*N*×(*C*/*n*),^ the query, key, and value matrices *Q*, *K*, and *V* are calculated as

(4) $$\begin{array}{c} Q=PW_{Q}\text{,}\\ K=PW_{k}\text{,}\\ V=PW_{v}\text{,} \end{array}$$

where *W*_*Q*_, *W*_*k*_, and *W*_*v*_ are the projection matrices. Generally, we have *Q*, *K*, *V* ∈ *ℝ*^*B*×*h*×*N*×(*C*/*n*)^. *h* is the number of heads. The original MHA directly uses *Q*, *K*, and *V* for large-scale matrix multiplication computations, resulting in a large amount of resource GPU memory and computational resources being occupied,. i.e., while calculating directly with *Q* and *K*, the shape of the self-attention matrix is *B* × *h* × *N* × *N* and then we perform matrix multiplication with *V*. However, hyperspectral images usually have high resolution, causing the *N* after dividing the patch to be very large. Obviously, direct calculations are not suitable for hyperspectral data.

In SR tasks, the predicted pixels of super-resolution images usually depend only on local neighbourhood in LR. However, the local neighbourhood is much larger than CNN's receptive field. The spatial and temporal complexity of the model can be reduced by dividing the feature into blocks. Hence, we use the feature segmentation module (FSM [31]) to divide *Q*, *K*, and *V* into *s* segments, where a segment is denoted by a triplet (*Q*_*i*_, *K*_*i*_, *V*_*i*_), *i*={1, ⋯, *s*}. The size of the local neighbourhood is controlled by *s*. Each segment performs a self-attention calculation separately and obtains the intrasegment self-attention matrix *O*_*i*_, which is thus computed by the self-attention mechanism in a segment as

(5) $$\begin{array}{c} O_{i}=Attention\left(Q_{i},K_{i},V_{i}\right)\\ =SoftMax\left(\frac{Q_{i}K_{i}^{T}}{\sqrt{d}}\right)V_{i}\text{.} \end{array}$$

We perform the attention function for *h* times in parallel and concatenate the results for multihead self-attention (MHA). Then, the results of each segment were combined to generate a complete attention matrix *O*. GPU memory usage is further reduced significantly by using segmentation for self-attention matrix computation.

(6) $$\begin{array}{c} 0=\left[O_{1},O_{2},\cdots ,O_{s}\right]\text{.} \end{array}$$

Next, a multilayer perceptron (MLP) with two fully connected layers and GELU nonlinearity between layers is used for further feature transformation. Layer Norm (LN) layers are placed before the MHA and MLP, and both modules are connected using residuals. Finally, to be consistent with the dimensions of the original input features, we restore it to its original dimensions by an expansion layer. The whole process is formulated as

(7) $$\begin{array}{c} P=MHA\left(Norm\left(P\right)\right)+P\text{,}\\ P=MLP\left(Norm\left(P\right)\right)+P\text{,} \end{array}$$

where *P* is denoted as the input to the efficient transformer block.

### 3.4. Spatial-Spectral Attention Module

**Figure 3:**
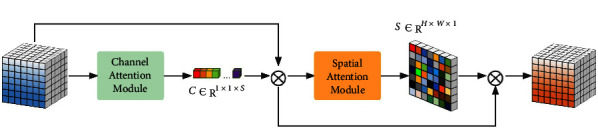
The architecture of the convolution block attention module.

Spectral characteristics are another important feature of HSI. Conventional convolution operations usually act on the entire waveband, which leads to spectral disorder and distortion. In addition, Transformer prefers to capture low-frequency information and lacks local high-frequency information. To address these problems, we use CBAM [32] (see Figure 3) to act as a spatial-spectral attention module to refine and correct the features. In detail, the weight descriptor of each channel is calculated along the space dimension and then multiplied with the channel of the corresponding feature map *F* to make it consistent with the GT spectral feature, which plays an important role in correcting the spectrum effect. Next, spatial weight descriptors are computed along the spectral dimension and multiplied by the pixels at each corresponding location to enhance important regions of the image, such as edges. The specific calculation steps are as follows:

(8) $$\begin{array}{c} CA=\sigma \left(MLP\left(AvgPool\left(F^{s}\right)+MLP\left(MaxPool\left(F^{s}\right)\right)\right)\right)\text{,}\\ SA=\sigma \left(MLP\left(AvgPool\left(F^{c}\right)+MLP\left(MaxPool\left(F^{c}\right)\right)\right)\right)\text{,} \end{array}$$

where *F*^*s*^ and *F*^*c*^ represent compression along the spatial dimension and compression along the channel dimension, respectively, and AvgPool and MaxPool represent average and maximum pooling operations, respectively. *σ* is the sigmoid operation.

### 3.5. Long Residual Connection

**Figure 4:**
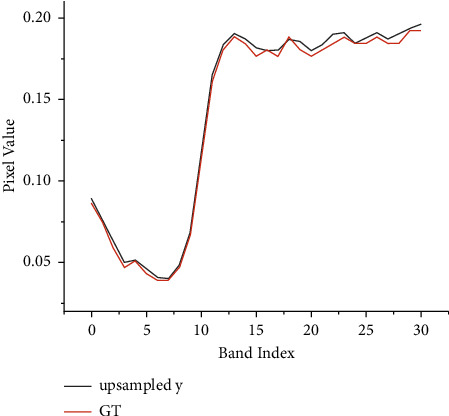
Spectral features of flowers at (250, 250) for GT and up-sampled *y*^*u*^.

As shown in Figure 4, upsampled LR-HSI *y*^*u*^ and GT HR-HSI *x* ∈ *ℝ*^*H*×*W*×*S*^ have the same number of bands, and most of the spectral information of HR-HSI is contained in upsampled LR-HSI. We plot the spectral vectors of GT and *y*^*u*^ at a location in Figure 4 to confirm it. Therefore, in order to maximize the retention of spectral information, *y*^*u*^ is passed to the end of the network through a long residual connection to directly sum up with the output features of another part of the network ∈*ℝ*^*H*×*W*×*S*^, followed by a 3 × 3 convolution for spatial-spectral feature adaptive fusion, finally generating the final high-resolution hyperspectral image *I*. The remaining spectral information is acquired by another part of the network. In addition, *y*^*u*^ contains more low-frequency information, and transmitting it directly to the end makes the network focus on learning high-frequency information, reducing the pressure on the network to reconstruct the whole HR-HSI.

### 3.6. Loss Function

In order to measure the super-resolution performance, several cost functions are studied to make the super-resolution result close to the real high-resolution image of the ground. In the current literature, *L*_2_ and *L*_1_ are the most used and relatively reliable loss functions. Compared with *L*_1_ loss, the MSE loss function is beneficial to the peak signal-to-noise ratio (PSNR), but it has several limitations, such as convergence and excessive smoothing. Therefore, we use *L*_1_ loss to measure the accuracy of network reconstruction. Where *L*_1_ loss is defined by Mean Absolute Error MAE (MAE) between all reconstructed images and real ground values

(9) $$\begin{array}{c} L_{1}=\frac{1}{N}\overset{N}{\underset{i=1}{\sum }}{\left\|o^{i}-x^{i}\right\|}_{1}\text{,} \end{array}$$

where the superscript *i* denotes the ith out of the *N* total training images.

The previous losses can well preserve the spatial information of the super-resolution results. However, the correlation between spectral features is ignored, and the reconstructed spectral information may be distorted. In order to ensure the spectral consistency of the reconstruction results at the same time, we apply a spectral angle loss function between the reconstructed image and the ground truth

(10) $$\begin{array}{c} L_{spec}=\frac{1}{NS}\overset{N}{\underset{i=1}{\sum }}\overset{S}{\underset{s=1}{\sum }}acrcos\left(\frac{o^{i,s}\cdot x^{i,s}}{{\left\|o^{i,s}\right\|}_{2}\cdot {\left\|x^{i,s}\right\|}_{2}}\right)\text{,} \end{array}$$

where the superscript *s* denotes the *s*th out of the *S* total spectral bands.

In summary, the final objective loss function used to optimize the model consists of the weighted sum of the previous two losses

(11) $$\begin{array}{c} L_{total}=L_{1}+\alpha \cdot L_{spec}\text{,} \end{array}$$

where *α* is used to balance the contributions of different losses. In our experiments, we set it as a constant, *α*=0.5.

## 4. General Information

### 4.1. Data Sets

We conduct a detailed analysis and evaluation of our proposed method with three public hyperspectral image datasets. These include two natural hyperspectral image datasets, the CAVE dataset [49] and one remote sensing hyperspectral image dataset, the Chikusei dataset [50].

**Figure 5:**
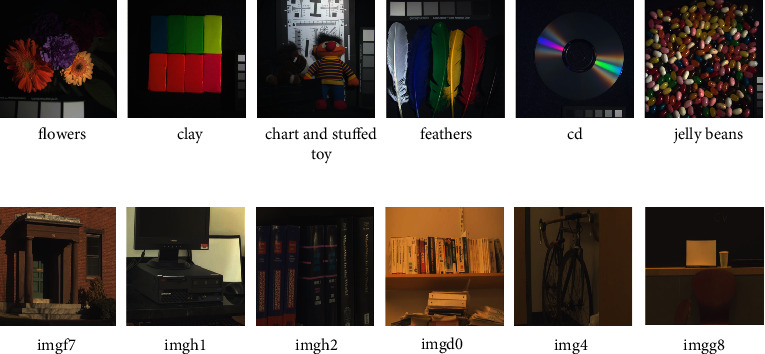
Some colour images from the CAVE dataset (a) and the Harvard dataset (b).

The abovementioned three data sets serve as ground truth values of high spatial resolution HR-HSI, whose corresponding HR-MSI is generated by using the corresponding camera spectral response function (SRF). The CAVE and Harvard datasets (see Figure 5) use Nikon D700, and the Chikusei datasets use Canon EOS 5D Mark II.

### 4.2. Comparison Methods

Five state-of-the-art hyperspectral super-resolution methods are selected as baselines for comparison with our proposed method. Among them, three are traditional fusion methods, namely, CSTF [16], FUSE [51], and GLP-HS [52] and the other two are deep learning fusion methods MHFnet [34] and HSRnet [26]. For a fair comparison, all comparison methods are from the public code. In addition, both HSRnet and MHFnet training datasets are consistent with this paper.

### 4.3. Evaluation Measures

Six quantitative image quality metrics widely used in image domains are used to comprehensively evaluate the performance of our proposed method, namely, Cross Correlation (CC), Spectral Angle Mapping (SAM) [53], Root Mean Square Error (RMSE), Relative Global Dimensional Synthesis Error (ERGAS) [54], Peak Signal-to-Noise Ratio (PSNR), and Structural Similarity (SSIM) [55]. PSNR and SSIM evaluate the spatial reconstruction quality of each band of the image. CC and SAM evaluate image spectral reconstruction quality. RMSE and ERGAS evaluate image loss size.

## 5. Results and Discussion

### 5.1. Performance on CAVE Dataset

The CAVE dataset contains 32 indoor HSI data under controlled lighting conditions with an image size of 512 × 512 and contains 31 bands ranging from 400 to 700 nm, with spectral bands in 10 nm steps.

For the CAVE dataset, 20 images are randomly selected from the CAVE dataset as a training set and the rest are used for testing. First, we normalize the dataset within the range of [0,1] and randomly crop each image to extract 3920 patches of size 64 × 64 × 31 as HR-HSI. Then, the HR-HSI bicubic downsampling (OpenCV-python function resize) is used to generate the corresponding LR-HSI patches, where the downsampling factor is 4. HR-MSI patches were generated by the Nikon D700 spectral response function identical to most of the experiments; 80% of the training set was used for training and 20% was the validation set.

**Figure 6:**
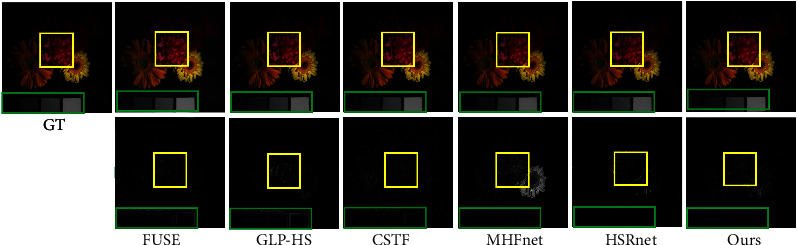
Column I: Pseudo-colour image of flowers (R-6, G-16, B-26). Columns II–VII: Pseudo-colour fused images of different methods and error maps, with some close-up boxes to facilitate visual analysis.

**Figure 7:**
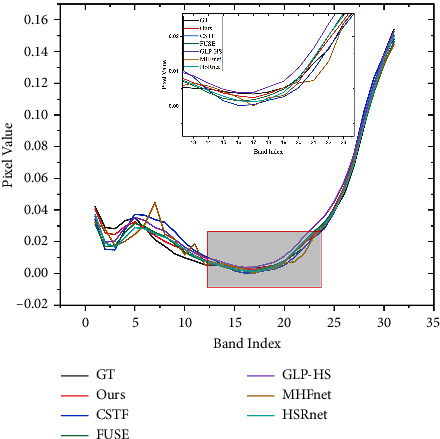
Spectral signature at position (250, 249).

We experimented directly with 11 test images with the trained model.  Table 1 gives the average test metric results for 11 test images under different methods. In order to let the reader have an intuitive feeling, we select a test image flower to present the fusion result pseudo-colour image and the corresponding error map in different ways.  Table 2 gives the indicators of different methods on the specified image. It is obvious that our method outperforms other comparative methods. As can be seen from the error diagram in Figure 6, the corresponding error of our proposed method is smaller than that of the comparison method, which shows that HMFT is more effective in recovering fine-grained texture and coarse-grained structure. On the contrary, it can be clearly seen that the fusion result of the HSRnet method has some obvious light spots, while the MHFnet image outline is still clearly visible, and the error is large. In addition, we plot the spectral vectors of the specified images to observe the spectral fidelity (see Figure 7). The spectral vectors of the fusion results of our method are most like those of GT.

### 5.2. Performance on Harvard Dataset

The Harvard dataset contains 50 indoor and 22 outdoor HSIs captured under daylight illumination. The spatial size is 1392 × 1040 with 31 bands in 10 nm steps covering the visible spectrum from 420 to 720 nm.

We select the upper left corner of the image (1000 × 1000) and then randomly select 10 images for testing. The same as the previous settings, the raw data are regarded as HR-HSI, and the acquisition method of LR-HSI and HR-MSI is the same as that of section B. We are consistent with the HSRnet approach, that is, without any retraining or fine-tuning of the models, we test them directly on the Harvard dataset, whose performance on the Harvard dataset can directly reflect the generalization ability of the model.

**Figure 8:**
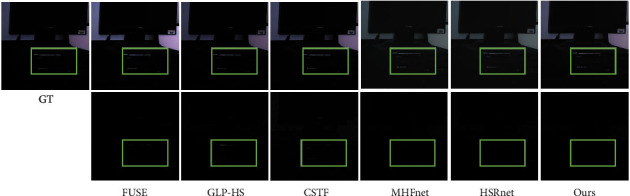
Computer (R-8, G-18, B-28) pseudo-colour image. Columns II–VII: pseudo-colour fusion results of different methods and corresponding error maps, and box-selected areas are convenient for visual analysis.

**Figure 9:**
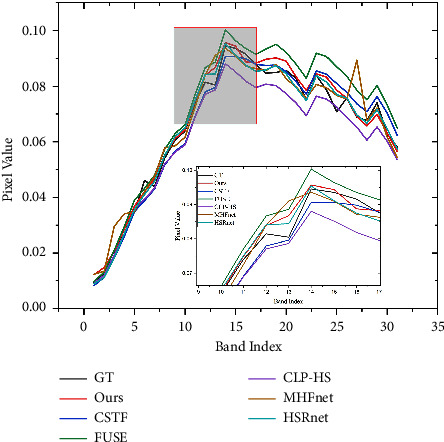
Spectral signature at position (238, 190).

The Harvard dataset has the same band as CAVE, no special training is performed, and 10 images are randomly selected for testing directly.  Table 3 gives the average indicator results for different methods. Likewise, we select a test image computer and plot the pseudo-colour images of the fusion results of different methods, the corresponding error maps, and spectral vectors.  Table 4 gives detailed metrics for different methods for specific images. From Figure 8, there is a significant colour difference in the fusion results of MHFnet and HSRnet. In addition, the ERGAS and SAM comparison index values of CSTF and MHFnet fluctuate significantly, indicating that the model is sensitive to the parameters of different images and has weak generalization ability. However, our proposed method is stable in all indicators, indicating that the model generalization ability is better than other methods.  Figure 9 also shows that our spectral fidelity is also better than other methods

### 5.3. Performance on Chikusei Dataset

In order to demonstrate the performance of our proposed method on hyperspectral remote sensing images, we conducted experiments on Chikusei dataset. The Chikusei dataset contains an airborne HSI, taken by a Visible and Near-Infrared (NIR) imaging sensor over agricultural and urban areas in Chikusei, Ibaraki, Japan. The hyperspectral dataset has 128 bands in the spectral range from 363 nm to 1018 nm, and the scene consists of 2517 × 2335 pixels.

Likewise, the original data are treated as HR-HSI, and the LR-HSI is simulated according to the previous experimental method. The HR-MSI is generated by the Canon EOS 5D Mark II spectral response function. After that, we select the 1024 × 2048 region in the upper left corner as training data and randomly crop 3920 overlapping patches with a size of 64 × 64 . Eight non-overlapping 512 × 512 patches were cropped from the remainder for test data.

**Figure 10:**
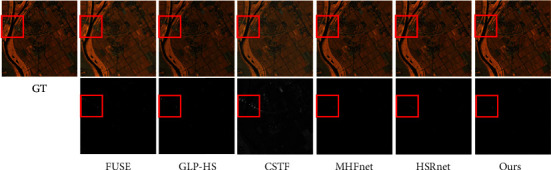
Column I: Pseudo-colour image of the 4th test image (R-31, G-56, B-89) of the Chikusei dataset. Columns II–VII: true false-colour fusion results for different methods and corresponding error maps, with some close-up boxes to facilitate visual analysis.

**Figure 11:**
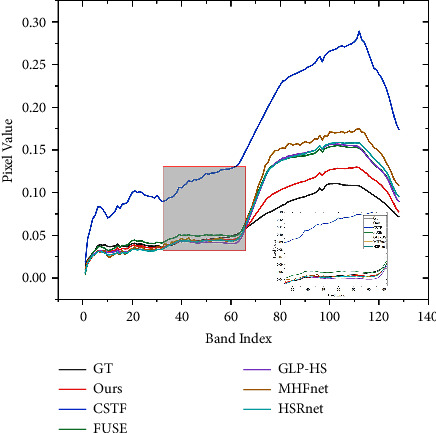
Spectral signature at position (250, 350).

Table 5 gives the average metrics on test data for different comparison methods. Clearly, our method outperforms other comparison methods on every metric. Likewise, we select an image to display its pseudo-colour image and the error map for visual comparison.  Table 6 gives the corresponding comparison index table. It can be clearly seen from Figure 10 that the fusion results of FUSE, GLP-HS, and CSTF are blurry and contain obvious spectral distortion. In addition, we also plot the spectral fidelity of the spectral features observed (see Figure 11). From a visual and data perspective, our method still performs well on hyperspectral remote-sensing images.

### 5.4. Ablation Study

*(1) Convolution Layer Analysis*. Transformer focuses more on global features and its self-attention mechanism can capture the global interactions between contexts well, while convolution focuses more on local features and can capture rich local details. We believe that the effective combination of the two can better learn the spatial-spectral information representation. To verify the validity of convolution, we compare our model with its variant, which has no convolutional layer. Tables 7 and 8 show the average metrics of the two networks on the CAVE dataset and the Harvard dataset. All indicators have been improved, so the network with convolution has a better effect.

*(2) Feature Split Module Analysis*. Hyperspectral images usually have high resolution, and direct calculation with native transformers will lead to huge computation and storage scale, and even more seriously, it may lead to memory overflow. In the SR task, we consider that the predicted pixels of the super-resolution image usually only depend on the local neighbourhood in the LR. Therefore, the same effect can be achieved by using the feature segmentation module (FSM) to block the features and then compute the attention. In order to demonstrate the effectiveness of the FSM, we conduct detailed comparative experiments on it.  Table 9 shows the average quality metrics of the two models on the CAVE dataset test images. Obviously, the network performance with the FSM module is better, especially the test time difference is 10 times, and the memory usage difference is 4 times, where the memory usage is obtained by the memory difference before and after the test module runs.

*(3) Convolutional Attention Mechanism Analysis*. In order to pay more attention to information features such as high-frequency information conducive to HR-HSI reconstruction and explore the correlation between spectra, we added a convolutional attention mechanism to further refine the extracted features in spatial and spectral dimensions, respectively. To demonstrate its effectiveness, we compare two networks with and without the convolutional attention mechanism. Among them, Table 10 shows the comparison results of 11 test images in the CAVE dataset. The network performance of the convolutional attention mechanism is relatively better.

## 6. Conclusions

In this paper, we propose a simple and efficient hyperspectral and multispectral fusion method. The network first uses convolution to propose shallow details and then uses Transformer to extract both spatial and spectral information of LR-HSI and HR-MSI. We added the FSM module to the MHA module of the Transformer to reduce the computational and memory costs. In addition, the CBAM module is added to the network to make more important channels and regions in the network. Finally, the L1 and spectral joint loss functions are used to train the whole network.

In future works, the HSI and MSI fusion method proposed in this paper will be further extended in two directions. On the one hand, multiscale technology is considered to further improve the feature extraction capability of the network. On the other hand, we try to further reduce the computational and memory cost of the model.

## Figures and Tables

**Table 1:** Average QIs and associated standard deviations for the results on 11 testing images on the CAVE data set.

Method	PSNR	SAM	ERGAS	RMSE	CC	SSIM
FUSE	46.57	5.26	1.96	1.4563	0.9976	0.9893
GLP-HS	44.45	7.60	2.34	1.8354	0.9968	0.9778
CSTF	44.78	5.04	2.19	1.6877	0.9975	0.9899
MHFnet	46.60	3.37	2.01	0.0060	0.9978	0.9920
HSRnet	47.25	3.00	1.54	0.0050	0.9989	0.9913
Ours	51.31	1.93	1.08	0.0032	0.9993	0.9959
Best value	+∞	0	0	0	1	1

The best values are underlined.

**Table 2:** QIs of the results by different methods on the specified flowers test image from the CAVE dataset.

Flowers (512 × 512 × 31)						
Method	PSNR	SAM	ERGAS	RMSE	CC	SSIM
FUSE	49.28	5.91	1.78	0.9656	0.9985	0.9933
GLP-HS	49.85	7.11	1.61	0.8759	0.9989	0.9916
CSTF	48.35	5.76	1.83	1.0001	0.9987	0.9931
MHFnet	52.11	3.80	1.33	0.0027	0.9993	0.9955
HSRnet	50.92	3.56	1.31	0.0029	0.9994	0.9933
Ours	56.29	2.20	0.74	0.0016	0.9997	0.9976
Best value	+∞	0	0	0	1	1

The best values are underlined.

**Table 3:** Average QIs for the ten test images on the Harvard data set.

Method	PSNR	SAM	ERGAS	RMSE	CC	SSIM
FUSE	37.88	3.33	5.73	4.3460	0.9817	0.9382
GLP-HS	47.82	2.71	2.37	1.2121	0.9960	0.9897
CSTF	45.72	9.95	3.20	1.5777	0.9921	0.9731
MHFnet	44.48	4.74	7.34	0.0066	0.9583	0.9771
HSRnet	48.63	2.66	2.34	0.0042	0.9963	0.9894
Ours	49.10	2.45	2.23	0.0040	0.9966	0.9977
Best value	+∞	0	0	0	1	1

The best values are underlined.

**Table 4:** QIs of the results by different methods on specified test computer images from the Harvard dataset.

Computer (512 × 512 × 31)						
Method	PSNR	SAM	ERGAS	RMSE	CC	SSIM
FUSE	36.72	2.71	8.8726	4.4762	0.9875	0.9705
GLP-HS	49.56	1.65	2.48	0.9116	0.9991	0.9932
CSTF	48.21	14.27	2.99	1.0664	0.9986	0.9835
MHFnet	47.18	4.48	7.58	0.0047	0.9628	0.9840
HSRnet	43.36	2.90	3.05	0.0074	0.9916	0.9770
Ours	52.08	2.78	2.17	0.0026	0.9996	0.9948
Best value	+∞	0	0	0	1	1

The best values are underlined.

**Table 5:** Average QIs of the results for eight testing images on the Chikusei dataset.

Method	PSNR	SAM	ERGAS	RMSE	CC	SSIM
FUSE	38.88	3.47	5.78	3.7769	0.9286	0.9157
GLP-HS	41.02	3.20	4.41	3.2109	0.9519	0.9313
CSTF	44.40	3.14	3.45	3.0527	0.9592	0.9324
MHFnet	44.48	4.74	7.34	0.0066	0.9583	0.9771
HSRnet	44.82	1.80	2.93	0.0090	0.9758	0.9653
Ours	45.30	1.71	2.78	0.0087	0.9779	0.9874
Best value	+∞	0	0	0	1	1

The best values are underlined.

**Table 6:** QIs of the results by different methods on specified 4th test images from the Chikusei dataset.

4th (512 × 512 × 31)						
Method	PSNR	SAM	ERGAS	RMSE	CC	SSIM
FUSE	39.88	3.28	5.53	3.6121	0.9305	0.9202
GLP-HS	42.36	2.76	4.03	2.9853	0.9567	0.9403
CSTF	46.10	2.39	2.98	2.3787	0.9711	0.9566
MHFnet	45.74	1.91	2.95	0.0085	0.9744	0.9662
HSRnet	46.29	1.65	2.67	0.0084	0.9782	0.9683
Ours	46.75	1.55	2.50	0.0081	0.9801	0.9701
Best value	+∞	0	0	0	1	1

The best values are underlined.

**Table 7:** Average QIs of the results on the CAVE and the Harvard data set using the proposed method HMFT with and without the convolution layer.

Method	PSNR	SAM	ERGAS	RMSE	CC	SSIM
w/o	49.53	2.64	1.19	0.0045	0.9962	0.9820
w	51.31	1.93	1.08	0.0032	0.9993	0.9959

The best values are underlined.

**Table 8:** Average QIs of the results on the Harvard data set using the proposed method HMFT with and without the convolution layer.

Method	PSNR	SAM	ERGAS	RMSE	CC	SSIM
w/o	48.98	2.79	2.23	0.0053	0.9964	0.9877
w	49.10	2.45	2.03	0.0040	0.9966	0.9877

The best values are underlined.

**Table 9:** Average QIs of the results and running time and memory usage on the CAVE dataset using the proposed method with and without the feature split module (FSM).

Method	PSNR	SAM	ERGAS	RMSE	Time	Memory
w/o	50.56	2.15	1.08	0.9959	1598.78	16.10
w	51.31	1.93	1.08	0.9959	152.50	4.03

The best values are underlined.

**Table 10:** Average QIs of the results on the CAVE dataset using the proposed method with and without the convolutional attention mechanism.

Method	PSNR	SAM	ERGAS	RMSE	CC	SSIM
w/o	49.17	2.07	1.08	0.0047	0.9948	0.9959
w	51.31	1.93	1.08	0.0032	0.9993	0.9959

The best values are underlined.

## Data Availability

The data that support the findings of this study are available on these websites (http://www.cs.columbia.edu/CAVE/databases/multispectral/, http://vision.seas.harvard.edu/hyperspec/download.html, http://naotoyokoya.com/Download.html).
